# Import of *Entamoeba histolytica* Mitosomal ATP Sulfurylase Relies on Internal Targeting Sequences

**DOI:** 10.3390/microorganisms8081229

**Published:** 2020-08-12

**Authors:** Herbert J. Santos, Yoko Chiba, Takashi Makiuchi, Saki Arakawa, Yoshitaka Murakami, Kentaro Tomii, Kenichiro Imai, Tomoyoshi Nozaki

**Affiliations:** 1 Department of Biomedical Chemistry, Graduate School of Medicine, The University of Tokyo, 7-3-1 Hongo, Bunkyo-ku, Tokyo 113-0033, Japan; hjsantos@m.u-tokyo.ac.jp; 2 Department of Parasitology, National Institute of Infectious Diseases, 1-23-1 Toyama, Shinjuku-ku, Tokyo 162-8640, Japan; yoko.chiba.ey@riken.jp (Y.C.); makky@tokai-u.jp (T.M.); 3 Graduate School of Life and Environmental Sciences, University of Tsukuba, 1-1-1 Tennodai, Tsukuba, Ibaraki 305-8572, Japan; saki.arakawa77@gmail.com (S.A.); yoym.gt@gmail.com (Y.M.); 4 Biofunctional Catalyst Research Team, RIKEN Center for Sustainable Resource Science (CSRS), Wako, Saitama 351-0198, Japan; 5 Department of Infectious Diseases, Tokai University School of Medicine, 143 Shimokasuya, Isehara, Kanagawa 259-1193, Japan; 6 Artificial Intelligence Research Center, National Institute of Advanced Industrial Science and Technology (AIST), 2-4-7 Aomi, Koto-ku, Tokyo 135-0064, Japan; k-tomii@aist.go.jp; 7 Cellular and Molecular Biotechnology Research Institute, National Institute of Advanced Industrial Science and Technology (AIST), 2-4-7 Aomi, Koto-ku, Tokyo 135-0064, Japan; kenichiro.imai@aist.go.jp

**Keywords:** mitosome, ATP sulfurylase, internal targeting sequence, *Entamoeba histolytica*, mitochondrion-related organelle, sulfate activation, reductive evolution

## Abstract

Mitochondrial matrix proteins synthesized in the cytosol often contain amino (N)-terminal targeting sequences (NTSs), or alternately internal targeting sequences (ITSs), which enable them to be properly translocated to the organelle. Such sequences are also required for proteins targeted to mitochondrion-related organelles (MROs) that are present in a few species of anaerobic eukaryotes. Similar to other MROs, the mitosomes of the human intestinal parasite *Entamoeba histolytica* are highly degenerate, because a majority of the components involved in various processes occurring in the canonical mitochondria are either missing or modified. As of yet, sulfate activation continues to be the only identified role of the relic mitochondria of *Entamoeba*. Mitosomes influence the parasitic nature of *E. histolytica*, as the downstream cytosolic products of sulfate activation have been reported to be essential in proliferation and encystation. Here, we investigated the position of the targeting sequence of one of the mitosomal matrix enzymes involved in the sulfate activation pathway, ATP sulfurylase (AS). We confirmed by immunofluorescence assay and subcellular fractionation that hemagluttinin (HA)-tagged *Eh*AS was targeted to mitosomes. However, its ortholog in the δ-proteobacterium *Desulfovibrio vulgaris*, expressed as *Dv*AS-HA in amoebic trophozoites, indicated cytosolic localization, suggesting a lack of recognizable mitosome targeting sequence in this protein. By expressing chimeric proteins containing swapped sequences between *Eh*AS and *Dv*AS in amoebic cells, we identified the ITSs responsible for mitosome targeting of *Eh*AS. This observation is similar to other parasitic protozoans that harbor MROs, suggesting a convergent feature among various MROs in favoring ITS for the recognition and translocation of targeted proteins.

## 1. Introduction

Two billion years of coevolution have established a complete co-dependence between the host archaeon and its endosymbiont of α-proteobacterial origin, presently known as the mitochondrion [1]. Cells depend on their mitochondria for a multitude of functions including aerobic respiration and metabolisms of carbohydrates, lipids, and amino acids [2]. Mitochondria contain DNA, however, almost all endosymbiont genes encoding mitochondrial proteins have been relocated to the host nuclear genome [3], thereby making them reliant on their host’s protein synthesis machinery which occurs in the cytosol. As a consequence, translocation mechanisms for mitochondrial proteins synthesized in the cytosol are essential. Such a process begins with the recognition of mitochondrial transport signal sequence contained in the protein [1]. The majority of known mitochondrial proteins possess an amino (N)-terminal sequence (NTS) called presequence, that acts as a transport signal for mitochondrial targeting and translocation. NTSs usually have high frequencies of arginine [4], lysine, and hydroxylated amino acids; low frequencies of aspartate and glutamate; and low hydrophobicity scores [5]. They are able to form positively charged amphipathic α-helices which are important for recognition by components of the translocase of the outer mitochondrial membrane (TOM) [6] such as Tom20 and Tom22 [7]. For matrix-targeted proteins, translocation, then, proceeds via crossing of the outer membrane through the TOM complex via Tom40 [8,9,10] and the presequence translocase (TIM23 complex) in the inner membrane [11]. The presequence translocase-associated motor (PAM) drives protein translocation into the matrix, where the mitochondrial processing peptidase (MPP) cleaves off the NTS [7,12,13,14]. However, not all mitochondrial proteins possess an NTS. Alternatively, these proteins are targeted through the recognition of a single or multiple internal targeting signal(s) (ITS) [15] by its receptor, Tom70 [1]. 

Reductive evolution and diversification of mitochondria into mitochondrion-related organelles (MROs) occurred in some eukaryotes that inhabit anaerobic or hypoxic niches as a consequence of oxygen-independent respiration and metabolisms [2]. Mitosomes constitute one of the five classes of mitochondria [16] and are present in a few anaerobic protozoan parasites such as *Entamoeba histolytica*, *Giardia intestinalis*, and *Cryptosporidium parvum*. A comparison with other MROs has shown that mitosomes retain the most minimal components and functions associated with canonical aerobic mitochondria because they lack genomic DNA, cristae structure, the electron transfer chain, and the ability to synthesize ATP [2,12]. In *E. histolytica*, mitosomes contain enzymes involved in the sulfate activation pathway, a process which usually takes place in the cytoplasm or plastids of other organisms [17]. ATP imported from the cytosol is used for the activation of sulfate by the action of three enzymes, ATP sulfurylase (AS), adenosine-5′-phosphosulfokinase (APSK), and inorganic pyrophosphatase [17]. This process yields activated sulfates such as 3′-phosphoadenosine-5′-phosphosulfate (PAPS) [17] utilized for the synthesis of sulfur-containing lipids in the cytosol [18].

Sulfate activation in *E. histolytica* mitosomes contributes to the parasitic and pathogenic nature of this organism [2]. *E. histolytica* causes human amebiasis, which is an intestinal disease that leads to diarrhea and dysentery. Worldwide, it causes up to 73,800 mortalities per year [19], with millions of people infected, primarily from ingestion of cysts from fecally-contaminated food or water. The resulting sulfolipids such as fatty alcohol disulfates have been reported to be indispensable to the survival of proliferative trophozoites [18], whereas cholesteryl sulfate has been linked to the induction of differentiation from trophozoites to cysts in the distantly related reptilian parasite *Entamoeba invadens* [17,20]. These findings underscore the role of sulfate activation in proliferation [18,21] and also in encystation [18,20]. The connected effects on crucial processes occurring in *E. histolytica* cells invoke the importance and contribution of mitosomes in maintaining its life cycle, as well as in disease transmission [2].

For sulfate activation to proceed, mechanisms for protein and metabolite translocation from the cytosol to the mitosomal matrix are required. However, *Entamoeba* mitosomes lack homologs for most of the components of the mitochondrial import complexes, except for the pore forming components Tom40 and sorting and assembly machinery 50 (Sam50), respectively [14]. In addition, not a single homolog for any component of the inner membrane import complex has been identified, yet several soluble proteins exist in the mitosomal matrix [17], suggesting the existence of novel import components that are most likely specific to its lineage. Proof of this is the import receptor Tom60, which is a TOM complex component present only in *Entamoeba* [22]. Furthermore, the mechanism of recognition and targeting of mitosomal proteins in *Entamoeba* remains unclear. In order to shed light on the protein targeting mechanisms in mitosomes, we focused on *Eh*AS, one of the matrix enzymes involved in sulfate activation. The gene encoding *Eh*AS is most likely acquired by lateral gene transfer [17]. Its amino acid sequence shares a high percentage identity (~60%) with that of a sulfate-reducing δ-proteobacterium, *Desulfovibrio vulgaris* (*Dv*). Both *Eh*AS and *Dv*AS lack predicted NTS, however the homolog in the free-living amoebozoan *Mastigamoeba balamuthi*, which also houses its sulfate activation pathway in another MRO called hydrogenosome, contains NTS [23]. On the basis of this information, we decided to search for the transport signal in the mitosome targeted *Eh*AS by expressing carboxy (C)-terminus hemagluttinin (HA)-tagged chimeric proteins containing swapped sequences from either *Eh*AS or *Dv*AS. Using these strains, we identified two ITS segments in *Eh*AS, suggesting divergent translocation mechanisms in the MROs of *Entamoeba* and *Mastigamoeba* for the matrix targeting of AS.

## 2. Materials and Methods

### 2.1. Cultivation of Entamoeba histolytica Cells

Trophozoites of *Entamoeba histolytica* clonal strain HM-1:IMSS Cl6 [24] were maintained axenically in Diamond’s BI-S-33 medium [24], as described previously. Cells were grown to the late-logarithmic phase (3–4 days after inoculation), prior to subculture.

### 2.2. Plasmid Construction

Total RNA was extracted from *E. histolytica* trophozoites, then, the mRNA was purified, and cDNA was synthesized following previously described protocols [25]. For expression in *E. histolytica* trophozoites with hemagglutinin (HA) tag at the C-terminus, the *E. histolytica (EhAS*, EHI_197160 [17]) gene was PCR-amplified from *E. histolytica* cDNA. The gene encoding *Desulfovibrio vulgaris* AS (*Dv*AS WP_010938590, codon optimized for *E. histolytica*) was synthesized by Eurofins Tokyo, Japan. These genes were inserted into the pEhExHA plasmid [26] cut with BglII (New England Biolabs, Beverly, MA, USA). *Eh*AS was divided into three blocks (block A, B, and C containing M1-F177, P178-V307, and G308-K423 of *Eh*AS, respectively) and each part was changed into the corresponding *Dv*AS sequences to make AS chimeras. The base parts, including pEhExHA and replacement parts (from homolog AS block), were PCR-amplified and ligated using In-fusion HD Cloning system (Takara, Shiga, Japan). To exchange smaller segments in block A and B, *Eh*AS-HA/pEhEx was PCR-amplified with primers phosphorylated at the 5′ end and containing partial *Dv*AS sequence. The amplified linearized plasmids were ligated using Ligation Convenience Kit (Nippongene, Chiyoda-ku, Tokyo, Japan). All primers used are listed in Table S1.

### 2.3. Amoeba Transformation

The plasmids generated as described above were introduced into *E. histolytica* trophozoites by lipofection, as previously described [27,28]. About 24 h after transfection, the medium was changed and 1 µg/mL of G418 (Gibco/Life Technologies, Waltham, MA, USA) was added to initiate selection. The concentration of G418 added was gradually increased for up to 2 weeks until it reached 10 µg/mL.

### 2.4. Immunoflourescence Assay (IFA)

As described previously [29], IFA of amoebic trophozoites was performed by double staining with anti-HA monoclonal antibody (clone 11MO, Covance, Princeton, NJ, USA) to detect hemagluttinin (HA)-tagged proteins and anti-adenosine-5′-phosphosulfate kinase (APSK) (EHI_179080, a mitosomal matrix protein) polyclonal rabbit antiserum [21] to label mitosomes. Cells that expressed HA-tagged proteins were analyzed according to the following three localization categories: mitosome, mitosome and cytosol, and cytosol. Briefly, cells categorized to have mitosome localization of respective HA-tagged proteins only show punctate signals that have good overlap with the mitosome marker. Dual localization to mitosomes and cytosol is characterized by a relatively uniform anti-HA staining of the cytosol with occasional puncta that colocalize with anti-APSK, whereas in purely cytosolic localization, such punctate anti-HA signals are absent.

### 2.5. Subcellular Fractionation and Immunoblot Analysis

Cells were grown to the late-logarithmic phase, harvested by the addition of ice-cold SM buffer (250 mM sucrose and 10 mM MOPS-KOH (pH 7.4)) to the culture flasks, and after discarding the medium, collected by centrifugation at 500× *g*, for 5 min, at 4 °C. The collected cells were resuspended with 1.0 mL of SM buffer with E-64 (Peptide Institute, Ibaraki-shi, Osaka, Japan) and Complete Mini EDTA-free (Roche Applied Science, Mannheim, Germany) protease inhibitors. Then, 0.1 mL of the resuspended cell pellet was aliquoted and TritonX-100 (final concentration 0.2%) was added. The sample was kept on ice for 10 min, centrifuged at 20,000× *g* for 10 min, at 4 °C, and the supernatant was named as total lysate. The remaining resuspended cells were disrupted using a Dounce homogenizer. Unbroken cells, nuclei, and large vacuoles were removed by centrifugation at 5000× *g,* at 4 °C, for 10 min. The supernatant was re-centrifuged at 100,000× *g* at 4 °C, for 1 h, and the supernatant was defined as cytosolic fraction. The pellet fraction was washed with SM buffer and recollected by centrifugation at 100,000× *g* at 4 °C, for 1 h. The resulting pellet was lysed with 100 μL of SM buffer with 1% of SDS (10 min on ice), followed by centrifugation at 20,000× *g* for 10 min, at 4 °C. The supernatant was defined as organelle fraction. The three fractions collected (total lysate, cytosol, and organelle) were run in SDS-PAGE followed by immunoblot analysis, as previously described [30]. The PVDF membranes were stained with anti-HA antibody, anti-APSK antiserum (organelle fraction marker), and anti-cysteine synthase 1 (CS1, cytosolic fraction marker EHI_171750, an enzyme involved in sulfur-containing amino acid metabolism) [31] and chemiluminescent bands visualized using a LAS-4000 mini luminescent image analyzer (Fujifilm Life Science, Minato-ku, Tokyo, Japan)

### 2.6. Structural Analysis

The three-dimensional structure of *Eh*AS was constructed by MODELLER [32] based on the alignment with AS from *Penicillium chrysogenum* (PDB ID: 1I2D) [33], calculated by FORTE, [34] except the N-terminal eight residues, since this portion can be a disordered region. 

## 3. Results

### 3.1. Wildtype EhAS-HA Is Localized to Mitosomes

**Figure 1:**
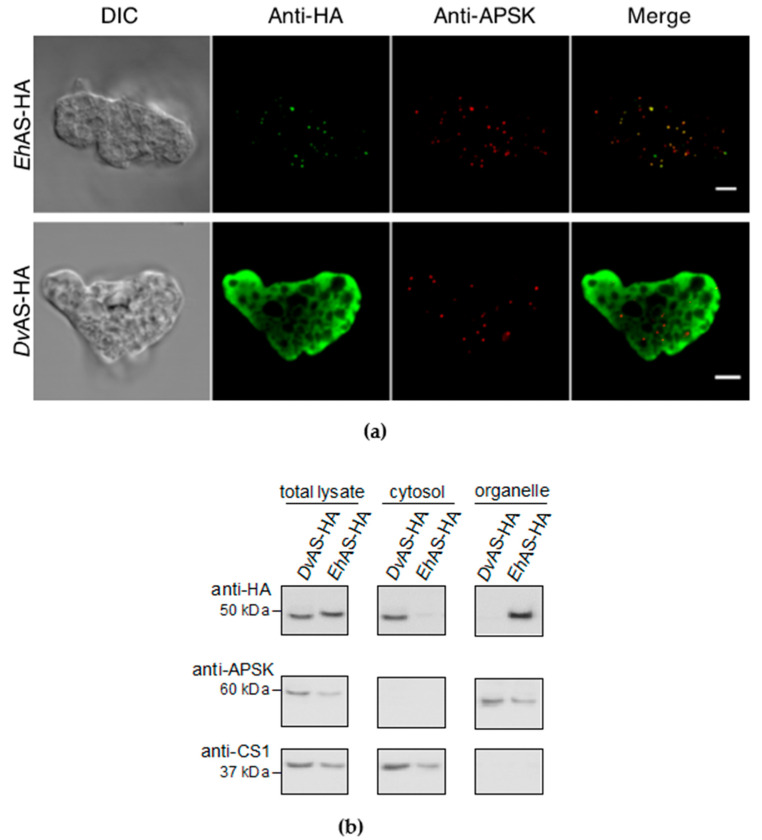
(**a**) Representative immunofluorescence assay (IFA) micrographs of wildtype C-terminal hemagluttinin (HA)-tagged *Eh*AS (top) and *Dv*AS (bottom) expressed in *E. histolytica* trophozoites, double stained with anti-HA antibody (green) and anti-APSK antiserum (red), respectively. Scale bar, 5 µm and DIC, differential interference contrast; (**b**) Immunoblotting profiles of the total lysate, cytosol, and organelle fractions of *Dv*AS-HA and *Eh*AS-HA, respectively. Membranes were stained with anti-HA antibody (top panel), anti-APSK (organelle marker, middle panel), and anti-CS1 antisera (cytosol marker, bottom panel), respectively.

Carboxy-terminal HA-tagged wildtype *Eh*AS-HA was expressed in amoebic trophozoites. The results of the immunofluorescence assay (IFA) indicate that *Eh*AS-HA is localized to mitosomes, as reported previously [17]. This is evidenced by strong colocalization between the green anti-HA signal with the red anti-APSK signal used as a mitosome marker (Figure 1a). Mitosome localization of *Eh*AS-HA was confirmed in all 96 cells observed (Table 1), We also performed subcellular fractionation of *Eh*AS to validate our imaging results. Figure 1b shows the immunoblot profiles following fractionation, clearly demonstrating that *Eh*AS-HA (right lane, top panel) is predominantly detected in the organelle fraction, similar to the fractionation staining profile of APSK (organellar marker, middle panel) and contrary to that of CS1 (cytosolic marker, bottom panel).

### 3.2. DvAS-HA Expressed in E. histolytica Was not Targeted to Mitosomes

In order to determine the localization of *Dv*AS in *E. histolytica* trophozoites, we performed similar IFA and subcellular fractionation, as discussed in the previous section. Double-staining IFA of *Dv*AS-HA showed cytosolic anti-HA signal (Figure 1a), distinctly different from the punctate anti-APSK signals, indicating that the protein was not translocated to mitosomes and remained in the cytosol. All 68 cells observed showed this localization to the cytosol (Table 1), indicating the absence of any targeting sequence in *Dv*AS that is recognizable by *E. histolytica*. Furthermore, these data were supported by immunoblotting analysis following subcellular fractionation, clearly showing *Dv*AS-HA is exclusively collected in the cytosolic fraction (Figure 1b).

### 3.3. Identification of EhAS Targeting Sequence by Constructing Chimeric Proteins with Swapped Blocks A, B, and C

**Figure 2:**
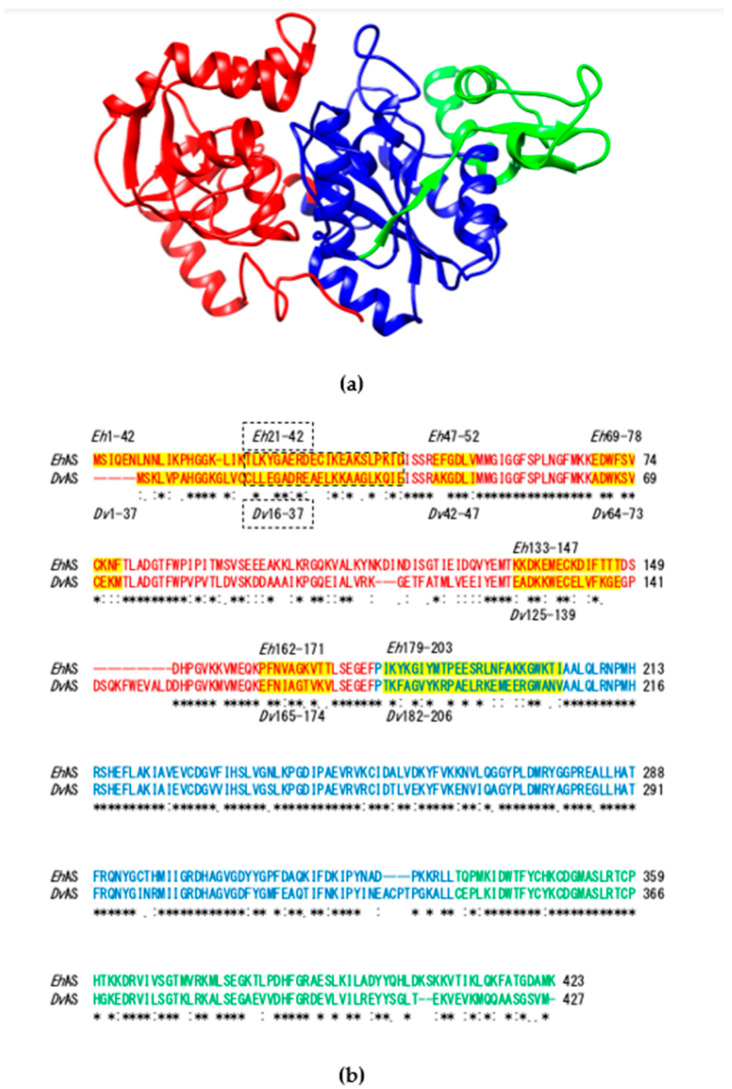

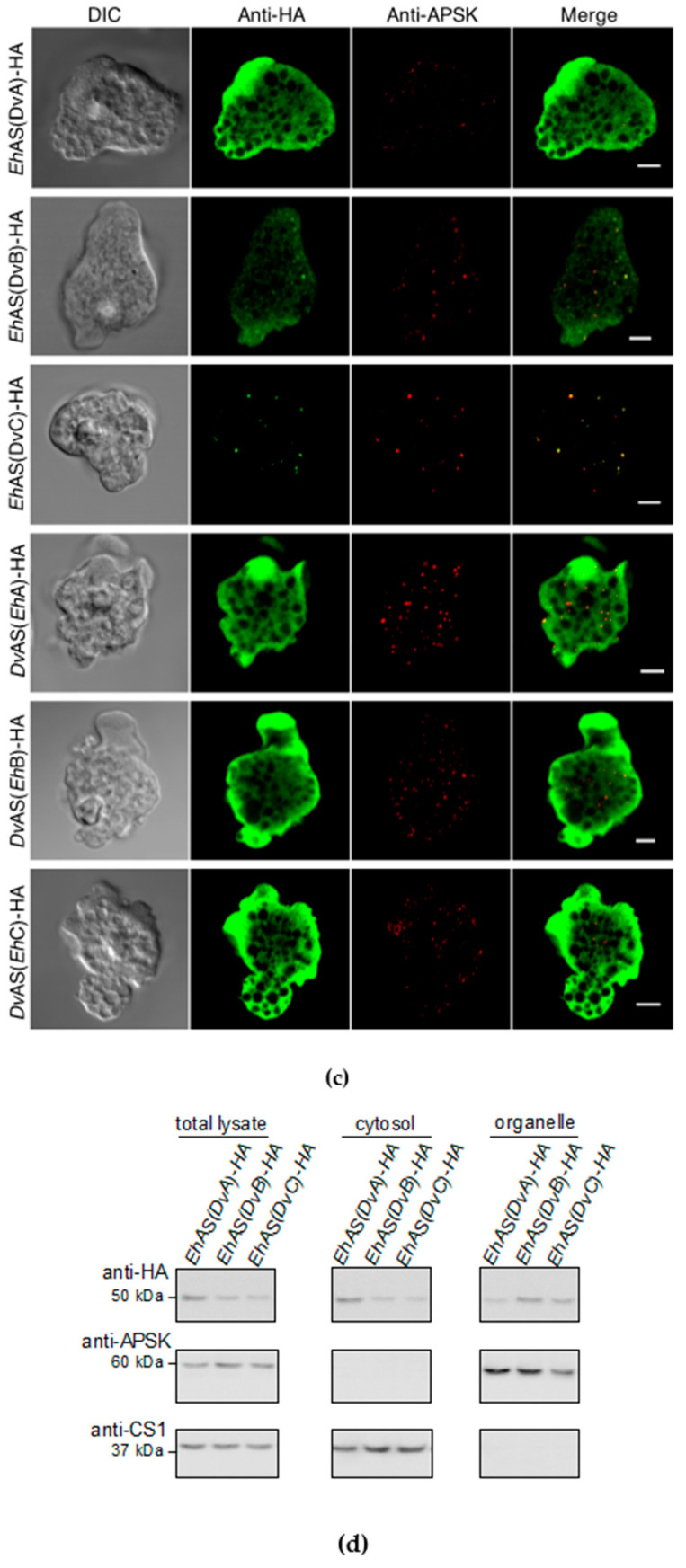
(**a**) Three-dimensional structure of *Eh*AS based on the alignment with AS from *Penicillium chrysogenum* were prepared with UCSF Chimera [35]. Ribbons depicting the three blocks A, B, and C are colored in red, blue, and green, respectively; (**b**) Amino acid sequence alignment of *Eh*AS and *Dv*AS using Clustal W [36] with the default parameters. The three major blocks A, B, and C are depicted in red, blue, and green text, respectively. Specific regions in block A and B are highlighted in yellow. *Dv*AS16–37 together with the corresponding *Eh*AS sequence is denoted with a dotted box to differentiate it from the overlap with *Dv*AS1–37; (**c**) Representative immunofluorescence assay (IFA) micrographs of chimeric *Eh*AS(*Dv*A)-HA, *Eh*AS(*Dv*B)-HA, *Eh*AS(*Dv*C)-HA, *Dv*AS(*Eh*A)-HA, *Dv*AS(*Eh*B)-HA, and *Dv*AS(*Eh*C)-HA expressed in *E. histolytica* trophozoites, double stained with anti-HA antibody (green) and anti-APSK antiserum (red) respectively. Scale bar, 5 µm; (**d**) Immunoblotting profiles of the total lysate, cytosol, and organelle fractions of chimeric *Eh*AS(*Dv*A)-HA, *Eh*AS(*Dv*B)-HA, and *Eh*AS(*DvC*)-HA, respectively. Membranes were stained with anti-HA antibody (top panel), anti-APSK (organelle marker, middle panel), and anti-CS1 antisera (cytosol marker, bottom panel), respectively.

To determine the position of the mitosomal targeting sequence in *Eh*AS, a few chimeric constructs based on either *Eh*AS or *Dv*AS were made. First, we divided the AS proteins into three major blocks based on the three-dimensional structure as depicted in Figure 2a. Chimeras using either *Eh*AS or *Dv*AS as base were designed by swapping sequences from respective blocks from either protein (Figure 2b). Using *Eh*AS as the backbone, we replaced its block A (M1-F177), block B (P178-L334), and block C (T335-K423), with the corresponding *Dv*AS sequences to yield *Eh*AS(*Dv*A), *Eh*AS(*Dv*B), and *Eh*AS(*Dv*C), respectively. Western blotting confirmed that all these chimeric proteins were expressed in trophozoites. The IFA data indicated that *Eh*AS(*Dv*A)-HA was localized to the cytosol (Figure 2b) in all 85 cells observed (Table 1). In contrast, *Eh*AS(*Dv*B)-HA was localized to mitosomes (Figure 2b) in 68% of expressing cells observed, with 14% showing signals in both mitosome and cytosol, and 18% displaying localization to cytosol (Table 1). *Eh*AS(*Dv*C)-HA, signals were almost exclusively colocalized with the mitosome marker (Figure 2b) in 94% of expressing cells observed (Table 1). These data suggest that replacement of block A and B in *Eh*AS, caused a decrease in the efficiency of transport to the mitosomes, and that the targeting signal(s) likely exist in these regions. These findings are supported by organelle fractionation followed by immunoblotting, as shown in Figure 2c. 

Conversely, we constructed chimeras where *Dv*AS block A (M1-F180), B (P181-L341), and C (C342-M427) were replaced with corresponding sequences from *Eh*AS and designated them as *Dv*AS(*Eh*A), *Dv*AS(*Eh*B), and *Dv*AS(*Eh*C), respectively. The IFA analysis revealed that 15% of observed cells that expressed *Dv*AS(*Eh*A)-HA showed mitosome and cytosol localization (Figure 2b and Table 1), whereas the remaining 85% showed cytosol localization. This suggests that *Eh*AS block A contains targeting sequence which allowed the *Dv*AS translocation, to a certain degree, to the mitosomes. On the contrary, *Dv*AS(*Eh*B)-HA (*n* = 156) and *Dv*AS(*Eh*C)-HA (*n* = 53) were exclusively localized to the cytosol (Figure 2b and Table 1), suggesting that block B and C of *Eh*AS alone were insufficient for the targeting of *Dv*AS to the mitosomes.

#### Refinement of AS Targeting Sequence Analysis in Blocks A and B

**Figure 3:**
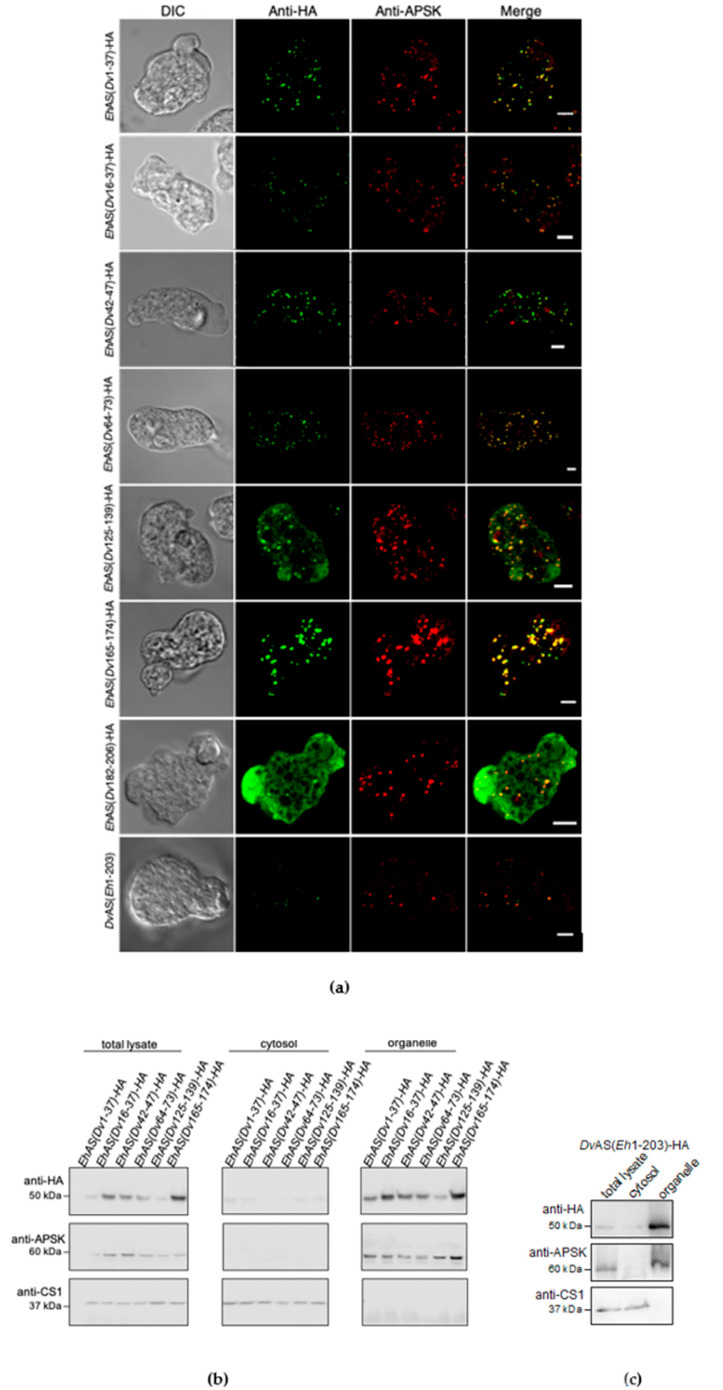
(**a**) Representative immunofluorescence assay micrographs of chimeric *Eh*AS(*Dv*1–37)-HA, *Eh*AS(*Dv*16–37)-HA, *Eh*AS(*Dv*42–47)-HA, *Eh*AS(*Dv*64–73)-HA, *Eh*AS(*Dv*125–139)-HA, *Eh*AS(*Dv*165–174)-HA, *Eh*AS(*Dv*182–206)-HA, and *Dv*AS(*Eh*1–203)-HA expressed in *E. histolytica* trophozoites, double stained with anti-HA antibody (green) and anti-APSK antiserum (red), respectively. Scale bar, 5 µm; (**b**) Immunoblotting profiles of the total lysate, cytosol, and organelle fractions of *Eh*AS(*Dv*1–37)-HA, *Eh*AS(*Dv*16–37)-HA, *Eh*AS(*Dv*42–47)-HA, *Eh*AS(*Dv*64–73)-HA, *Eh*AS(*Dv*125–139)-HA, *Eh*AS(*Dv*165–174)-HA, and (**c**) *Dv*AS(*Eh*1–203)-HA, respectively. Membranes were stained with anti-HA antibody (top panel), anti-APSK (organelle marker, middle panel), and anti-CS1 antisera (cytosol marker, bottom panel), respectively.

To narrow down the mitosome targeting sequence of *Eh*AS that is likely located in block A and B, we selected seven smaller regions which had lower amino acid sequence similarity between *Eh*AS and *Dv*AS than other regions. These were residues 1–42, 21–42, 47–52, 69–78, 133–147, 162–171, and 179–203 of *Eh*AS, which correspond to residues 1–37, 16–37, 42–47, 64–73, 125–139, 165–174, and 182–206 in *Dv*AS, respectively (Figure 3a). Then, we created constructs to express chimeric proteins using *Eh*AS as the base sequence, containing corresponding refined regions from the *Dv*AS sequence. IFA found almost no change in mitosome targeting of chimera *Eh*AS(*Dv*16–37) and *Eh*AS(*Dv*165–174) with 99% and 93% of observed cells, respectively, showing exclusive localization to mitosomes (Figure 3a), whereas 1% and 7%, respectively, had anti-HA signals in both mitosome and cytosol (Table 1). Meanwhile, a slightly decreased efficiency in the targeting of chimera *Eh*AS(*Dv*1–37), *Eh*AS(*Dv*42–47), and *Eh*AS(*Dv*64–73) showed 88%, 83%, and 77% of observed cells with mitosome colocalization, respectively (Figure 3a and Table 1). All the remaining observed cells showed mitosome and cytosol localization in these three chimeras except for a single cell in *Eh*AS(*Dv*1–37) which showed complete retention of the protein in the cytosol. Together, these data indicate that the replaced *Eh*AS sequences are not essential for mitosome targeting. However, for the chimera *Eh*AS(*Dv*125–139), only 39% of expressing cells showed exclusive localization to mitosomes, whereas 58% showed localization in both mitosomes and cytoplasm (Figure 3a and Table 1). This is indicative of a relatively poor mitosome transport efficiency of this protein, and points to the possibility that the region replaced (*Eh*133–147) is recognized as a targeting sequence. In addition, *Eh*AS(*Dv*182–206), a chimera that contains a replaced segment of block B, was localized in mitosomes among 45% of expressing cells, and in both mitosomes and cytosol among the remaining 55% of expressing cells (Figure 3a and Table 1). The results from subcellular fractionation, followed by immunoblotting corroborate the observed IFA localization of these chimeric proteins (Figure 3b).

From these findings, it is possible that multiple regions need to be recognized rather than being limited to one region, and that signals could be contained in other regions that were not initially replaced. We, then, designed a converse chimera combining block A and a specific region in block B (*Eh*179–203) of *Eh*AS to replace the sequence in *Dv*AS and designated it as *Dv*AS(*Eh*1–203). IFA results revealed that all cells observed to express the chimeric protein showed punctate signal similar to that of mitosomes, with 91% showing colocalization with anti-APSK signal (Figure 3a and Table 1). The remaining 9% of expressing cells also displayed punctate, non-cytosolic anti-HA signals that did not colocalize to that of APSK. This could reflect that either mitosomes are heterogenous in composition, or that this chimera is mislocalized to uncharacterized organelles. 

## 4. Discussion

In this study, we aimed to identify the targeting sequence in the mitosomal matrix protein ATP sulfurylase (AS), whose gene is apparently acquired from bacteria by lateral gene transfer. We expressed *Eh*AS and one of its closest bacterial homologs, *Dv*AS, with HA-tag at the C-terminus to avoid possible interference with N-terminus recognition, although they both lack predictable NTS. *Eh*AS-HA clearly localized to mitosomes, as reported before [17]. In contrast, *Dv*AS-HA expressed in *E. histolytica* trophozoites remained in the cytosol, despite having a relatively high (60%) amino acid sequence identity to *Eh*AS, suggesting that this protein completely lacks any recognizable sequence for targeting to mitosomes. The targeting of *Eh*AS to mitosomes occurs independently of a predictable NTS. The free-living amoeba *Mastigamoeba balamuthi* also contains sulfate activation enzymes in its MRO, called hydrogenosome. Interestingly, all three sulfate activation pathway enzymes, including *Mb*AS, contain predictable NTS ([23], KF927024, Figure S1). This suggests possible divergent import mechanisms in the two sulfate-activating MROs, although the role of the predicted NTSs in *Mastigamoeba* has yet to be clarified. 

Upon expression of various chimeric proteins in *E. histolytica* trophozoites, wherein regions of AS sequences are swapped between *Eh*AS and *Dv*AS, we were able to identify the important internal segments required for import of this protein to mitosomes. IFA was conducted in all transformants and localization was categorized into mitosomes, mitosomes and cytosol, and cytosol, respectively, according to the signal pattern and colocalization of the anti-HA antibody detecting AS chimera, with the mitosome marker anti-APSK. On the basis of the results of blocks A, B, and C swapping, it was revealed that *Eh*AS block A likely contained the targeting sequence, because replacement of *Eh*AS block A with *Dv*AS block A completely abolished delivery of this chimeric protein to mitosomes. In strong support for this, only the converse replacement of block A of *Dv*AS with that of *Eh*AS demonstrated mitosome targeting to a certain degree (Figure 2b). When *Eh*AS block A was further divided into smaller regions, and subsequently swapped with *Dv*AS regions, only chimeric *Eh*AS(*Dv*125–139)-HA showed a drastic reduction in mitosome targeting efficiency. Interestingly, the replacement of *Eh*AS block B to form *Eh*AS(*Dv*B)-HA demonstrated partially diminished targeting to mitosomes, as indicated by the decreased mitosome localization to 68%, and observance of mitosome/cytosol (14%) and cytosol (18%) localization. This suggests that block B also contains a portion essential to targeting, and that *Eh*AS possesses multiple ITSs distributed in both block A and B. This is supported by the sufficiency of *Eh*AS residue 1–203 to allow translocation of the otherwise cytosolic *Dv*AS, to the mitosomes (Figure 3a,c).

The majority of the proteins detected in the proteomic survey of *E. histolytica* mitosomes are without any predictable NTS [17]. Previously, the NTS-like segment of the mitosomal *Eh* chaperonin 60 (*Eh*Cpn60) was investigated. Deletion of this sequence led to the mistargeting of the protein to the cytosol [37]. However, neither luciferase nor GFP with the NTS-like segment inserted on their N-termini resulted in mitosome targeting of these cytosolic passenger proteins [38], suggesting the NTS-like segment of *Eh*Cpn60 alone is not enough for recognition and delivery to mitosomes. In another work, the mitosomal inner membrane phosphate channel (*Eh*PiC), despite not having a predictable NTS, was successfully imported into *Saccharomyces cerevisiae* mitochondria. This suggests the presence of ITS in *Eh*PiC that is recognizable even by aerobic mitochondria [14]. Taken together with these previous studies, our finding suggests a decreased dependence on NTS in favor of ITS in *E. histolytica* mitosomal protein targeting. This has been similarly reported in other organisms possessing MROs. In the intestinal protozoan parasite *G. intestinalis*, cysteine desulfurylase, chaperonin 60, and mitochondrial heat shock protein 70, lack NTS but possess ITS for their delivery to mitosomes [39,40,41]. In the sexually transmitted protozoan parasite *Trichomonas vaginalis*, thioredoxin reductase 2 utilizes an ITS for hydrogenosome translocation [42]. It has also been reported that other *Trichomonas* hydrogenosomal proteins contain sequences predicted as NTSs but are not essential for targeting, as NTS-deletion mutants of *Tv* pyruvate ferredoxin oxidoreductase, malic enzyme, ferredoxin 1, and iron-sulfur assembly protein 1 were successfully translocated into the hydrogenosomal matrix [43], suggesting that they also contain ITS for proper recognition and delivery to the organelle. Overall, these findings indicate the presence of an ITS-dependent protein import system in various MROs, as well as in aerobic mitochondria.

A few possible factors exist that contribute to the selective pressure against an NTS-based import among MROs. One is the partial or complete loss of the components of the electron transport chain. The absence of a membrane potential generated by the electron transport chain makes the NTS dispensable as the electrophoretic force that drives protein transport mediated by the positively charged NTS residues across the inner membrane [15,44]. This may have influenced the eradication of any predictable NTSs in various MRO-targeted proteins in different organisms. The length of predicted NTSs in *T. vaginalis* and *G. intestinalis* (4 to 21 amino acids [45]) is relatively shorter than those of mitochondrion-targeted counterparts (15 to 50 amino acids [44]). NTSs in MROs also either completely lack or have reduced net positive charge as compared with mitochondrial-type NTSs [44]. Furthermore, abolishment of the membrane potential could have triggered a domino effect that enabled a restructuring of import complexes in MROs. For instance, the loss of the electrochemical gradient-dependent inner membrane complexes, Tim23 and Tim22 [15], could have influenced a restructured TOM complex. Tom22, which is also a receptor of the NTS [1], is an integral component of the TOM complex that interacts with the Tim23 complex via its intermembrane space domain [46]. Most, if not all, of these components are missing in MROs. In the case of *Entamoeba*, homologs of Tom22, as well as the other targeting sequence receptors Tom20 and Tom70, are absent in the genome. Instead, this parasite utilizes the lineage-specific receptor Tom60, acting as a receptor of both soluble and membrane proteins needed to be imported to the mitosomes [22]. 

Another factor that could have led to the shift in targeting sequence preference is the partial or complete loss of the MPP complex components that cleave NTS after protein translocation. Although the MROs of *T. vaginalis* [45,47], *C. parvum* [48,49], and *Blastocystis hominis* [50] contain both MPPα and MPPβ subunits, others such as *G. intestinalis* [45] and *E. histolytica* [12] only possess the catalytic MPPβ subunit. This appears to be sufficient in *Giardia* as the monomeric MPP could operate the cleavage of NTS [45]. Interestingly, *Eh*MPPβ is not localized to mitosomes but in the cytosol [12], while *Gi*MPPβ exists in the mitosomes [39]. This localization change could have been influenced by the shift in the reliance on ITSs from NTSs in this parasite, due to the loss of membrane potential on *E. histolytica* mitosomes, thereby making NTS processing using MPP in the matrix unnecessary. Other organisms such as the microsporidion *Encephalitozoon cuniculi* and the diplomonad *Spironucleus salmonicida* lack the genes for both MPP subunits [51,52,53]. It has been demonstrated that the matrix-targeted *E. cuniculi* glycerol-3-phosphate dehydrogenase remained functional despite having an uncleaved predicted NTS [52], suggesting the non-essentiality of MPP in this organism. Meanwhile, in *S. salmonicida,* all hydrogenosome-targeted proteins lack any predictable NTS [53] making the MPP complex superfluous.

The identification of two ITS segments located separately in *Eh*AS is crucial for elucidating the import mechanisms in *Entamoeba* mitosomes. In vitro, Tom60 has been demonstrated to bind *Eh*AS, as well as the “EEVD” motif of cytosolic Hsp70 and Hsp90, presumably via its tetratricopeptide repeats [22]. Although the binding of mitosomal proteins to Tom60 is not homologous to the charge-dependent interaction of NTS to canonical mitochondrial receptors, we noticed that the ITSs of *Eh*AS (133–147:KKDKEMECKDIFTTT and 179–203:IKYKGIYMTPEESRLNFAKKGWKTI) share similar features with NTSs. This has also been reported in yeast, after in silico analysis of yeast mitochondrial proteins revealed that their ITSs possess a number of characteristics that mimic NTS [5]. A comparison of primary structures of yeast matrix proteins revealed that ITSs have high frequencies of arginine, lysine, and hydroxylated amino acids, and low frequencies of aspartic acid and glutamic acid. In addition, ITS secondary structure is also predominantly helical and amphipathic with low hydrophobicity scores [5]. In fact, both *Eh*AS ITSs mainly consist of α-helices, and the lysine and arginine residues in both segments are located on the surfaces of those helices, although two glutamic acid residues are also located on the surface of the helix 188–198:PEESRLNFAKK (Figure S2). These also support that the two identified ITS segments can be critical to the targeting of *Eh*AS to mitosomes. 

The exact mechanism by which Tom60 recognizes and binds soluble mitosomal proteins such as *Eh*AS remains unknown. In the fungus *Neurospora crassa*, mitochondrial heme lyases lack NTS, however, their ITSs provide information that determines the specificity of transport into the intermembrane space. The import reaction is said to be driven by the high-affinity interaction between the ITS and import components of the intermembrane space [6]. Such mechanisms could also exist in *E. histolytica* mitosomes. However, many components of the import machinery, particularly at the intermembrane space, and the inner membrane remain undiscovered due to the lack of obvious sequence homology with other organisms. Thus, it is extremely difficult to paint a clearer picture of the import process occurring in *Entamoeba* mitosomes. In *G. intestinalis*, a metamonad-specific hidden Markov model-based search yielded a highly divergent homolog of the pore-forming Tim17, which interacts with *Gi*Tim44, a tethering molecule linking the Tim and Pam complexes [54]. Similar to *Giardia*, it would not be surprising if future studies reveal a minimalist mechanism of protein targeting and import in this relic mitochondria of *Entamoeba* that is accompanied by components unique to its genus. This is supported by discoveries of several *Entamoeba-*specific mitosomal membrane proteins [55] such as the β-barrel outer membrane protein MBOMP30 [25], and the transmembrane protein ETMP30 [29]. 

## 5. Conclusions

We identified two segments in *Eh*AS, (133–147:KKDKEMECKDIFTTT and 179–203:IKYKGIYMTPEESRLNFAKKGWKTI) which influence its targeting to mitosomes. The identification of mitosomal ITSs contributes to the understanding of the evolution of protein transport systems in MROs. NTS-independent recognition and translocation is a conserved feature among aerobic and reduced mitochondria, suggesting that the ancestral mode of protein targeting does not require NTS [56]. It appears that although NTS enhances specificity of targeting to the matrix, it is a non-essential additional factor for protein import, promoted by membrane potential generated by the electron transport chain at the inner membrane [44,56]. The loss of membrane potential first triggered reduction of the net positive charge, and eventually the complete removal of unnecessary NTS in mitochondrial proteins, followed by the loss of essentiality of MPP processing in the mitochondrial matrix. Other important unresolved issues of *Entamoeba* mitosomal protein import include the identification of protein import components, particularly in the intermembrane space and inner membrane. Most likely, these proteins are unique to *Entamoeba* because homologs have not been identified from currently available sequence databases. One example is the novel lineage-specific mitosome import receptor Tom60 [22]. Another issue is the loss of electrophoretic force in reduced mitochondria. What is used as a substitute for the electrophoretic force in driving the translocation of mitosomal matrix proteins? 

*Entamoeba* mitosomes have lost many associated functions of the canonical aerobic mitochondria. During the course of its adaptation to an anaerobic environment, its mitosomes have acquired new functions that directly influenced its parasitic and pathogenic lifestyle. Its protein import system also displayed reductive evolution, and the lost components were likely replaced by lineage-specific functional homologs. The identification of mitosome transport signals in *Eh*AS could lead to the unraveling of the entire *Entamoeba* mitosomal protein import machinery, including the existence and function of complexes that have not yet been discovered. Potential unique features of this organelle which is linked to the parasitic nature of *Entamoeba* [2], would be beneficial for a better understanding of its biology. Moreover, the discovery of novel *Entamoeba*-specific components of the import complexes in mitosomes could lead to the identification of new candidate targets for drug development against amebiasis. With such information, we could screen for inhibitors that would obstruct mitosomal translocation of sulfate activation enzymes, resulting in either death or failure of the parasite to undergo encystation.

## Figures and Tables

**Table 1:** Percentage of cells expressing various HA-tagged proteins that display mitosome, mitosome and cytosol, and cytosol localization.

Strain	% of Cells Showing Localization of HA-Tagged Protein to			*n*
	Mitosome	Mitosome and Cytosol	Cytosol	
*Eh*AS-HA 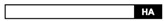	100	0	0	96
*Dv*AS-HA 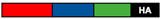	0	0	100	68
*Eh*AS(*Dv*A)-HA 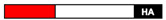	0	0	100	85
*Eh*AS(*Dv*B)-HA 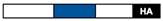	68	14	18	85
*Eh*AS(*Dv*C)-HA 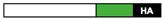	94	6	0	98
*Dv*AS(*Eh*A)-HA 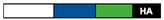	0	15	85	206
*Dv*AS(*Eh*B)-HA 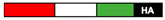	0	0	100	156
*Dv*AS(*Eh*C)-HA 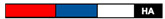	0	0	100	53
*Eh*AS(*Dv*1–37)-HA 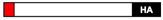	88	11	1	127
*Eh*AS(*Dv*16–37)-HA 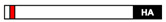	99	1	0	80
*Eh*AS(*Dv*42–47)-HA 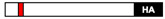	83	17	0	121
*Eh*AS(*Dv*64–73)-HA 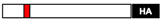	77	23	0	62
*Eh*AS(*Dv*125–139)-HA 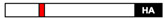	39	58	3	62
*Eh*AS(*Dv*165–174)-HA 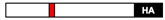	93	7	0	60
*Eh*AS(*Dv*182–206)-HA 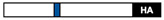	45	55	0	191
*Dv*AS(*Eh*1–203)-HA 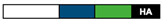	91 *	0	0	58

* The remaining 9% of observed *Dv*AS(*Eh*1–203)-HA expressing cells did not show colocalization between anti-HA and anti-APSK signals.

## Supplementary Materials

The following are available online at https://www.mdpi.com/2076-2607/8/8/1229/s1, Table S1: List of primer sets used in this study, Figure S1: Multiple sequence alignment of ATP sulfurylase of various *Entamoeba* species namely, *Entamoeba histolytica*, *E. nuttalli*, *E. dispar*, *E. moshkovskii*, and *E. invadens*, as well as that of *Acanthamoeba castellanii*, *Mastigamoeba balamuthi*, and *Desulfovibrio vulgaris*, Figure S2: Structural features of *Eh*AS internal targeting sequences.
