# Supplementary material for: Import of Entamoeba histolytica Mitosomal ATP Sulfurylase Relies on Internal Targeting Sequences
Source: Microorganisms. 2020 Aug 12;8(8):1229. doi: 10.3390/microorganisms8081229 (PMC7465240; doi:10.3390/microorganisms8081229)
Supplement: Supplementary file 1 [file microorganisms-08-01229-s001.pdf]

microorganisms

Supplementary Materials to

Import of *Entamoeba histolytica* mitosomal ATP sulfurylase relies on internal targeting sequences

Herbert J. Santos[1,2,3], Yoko Chiba[2,3,4], Takashi Makiuchi[2,5], Saki Arakawa[3*], Yoshitaka Murakami[3*], Kentaro Tomii[6], Kenichiro Imai[7], and Tomoyoshi Nozaki[1,2,3]**

Table S1. List of primer sets used in this study.

Plasmid name	Orientation	Sequence (5' - 3')
EhAS-HA	sense	ACAAACACATTAACAGATCTATGAGCATTCAAGAAAACTTAAAC
	antisense	TCATATGGATACATAGATCTTTTCATGGCATCACCAGTAGC
EhAS(DvA)-HA	sense	ATGTCAAAGTTAGTTCCAG
	antisense	TTCACCTTCTGATAAACTTTAAC
*Dv*AS(*Eh*A)-HA	sense	TTATCAGAAGGTGAATTTCCAATT
	antisense	AACTAACTTTGACATGTTTTCTTGAATGCTCATAGA
*Eh*AS(*Dv*B)-HA	sense	TTTCCAACAAAGTTTGCAG
	antisense	TCTACCTATAATCATACGATTAAT
*Dv*AS(*Eh*B)-HA	sense	ATGATTATAGGTAGAGATCATGCT
	antisense	AAACTTTGTTGGAAATTCTCCTTCTGAAA
*Eh*AS(*Dv*C)-HA	sense	GGAGTTGGAGATTTCTAT
	antisense	TCATATGGATACATTACTGAACCTGATGCA
*Dv*AS(*Eh*C)-HA	sense	TCAGGTTCAGTAATGTATCCATATGATGTTCCAGA
	antisense	GAAATCTCCAACTCCAGCAT
*Eh*AS(*Dv*1-37)-HA	sense	(phos)TAAAAGACAACAAACAAGTCCTTTTCCTCCATGAGCTGGAACTAAC TTTGACATAGATCTGTTAATGTGTTTGTCAGTTCA
	antisense	(phos)TTTTCCTCCATGAGCTGGAACTAACTTTGAAGATCTGTTAATGTGGTT TC
*Eh*AS(*Dv*16-37)-HA	sense	(phos)TTAAAAAAAGCTGCTGGTCTTAAACAAATAGAGATTTCATCACGAG AATTTGG
	antisense	(phos)TTCAGCTTCTCTATCAGCTCCTTCTAAAAGACATTTAATGAGTTTTCC TCCATG
*Eh*AS(*Dv*42-47)-HA	sense	(phos)GACTTAATTATGATGGGAATTGGAGG
	antisense	(phos)TCCTTTTGCTCGTGATGAAATATCAATC
*Eh*AS(*Dv*64-73)-HA	sense	(Phos)GTTTGTGAAAAAATGACACTTGCTGATGGAACA
	antisense	(phos)AGATTTCCAATACGCTTTTTTCATAAATCCATTTAATGG
*Eh*AS(*Dv*125-139)-HA	sense	(phos)TGAGTTAGTATTTAAAGGAGAATCAGACCATCCAGG
	antisense	(phos)CATTCCCATTTCTTGTCTGCTTCCTTAGTCATTTCATAAACTTGG
*Eh*AS(*Dv*165-174)-HA	sense	(phos)GAACAGTTAAAGTTCTTTCAGAAGGAGAATTTC
	antisense	(phos)CTGCAATGTTAAATTCTTTTTGTTCCATAACTTTTTTAAC

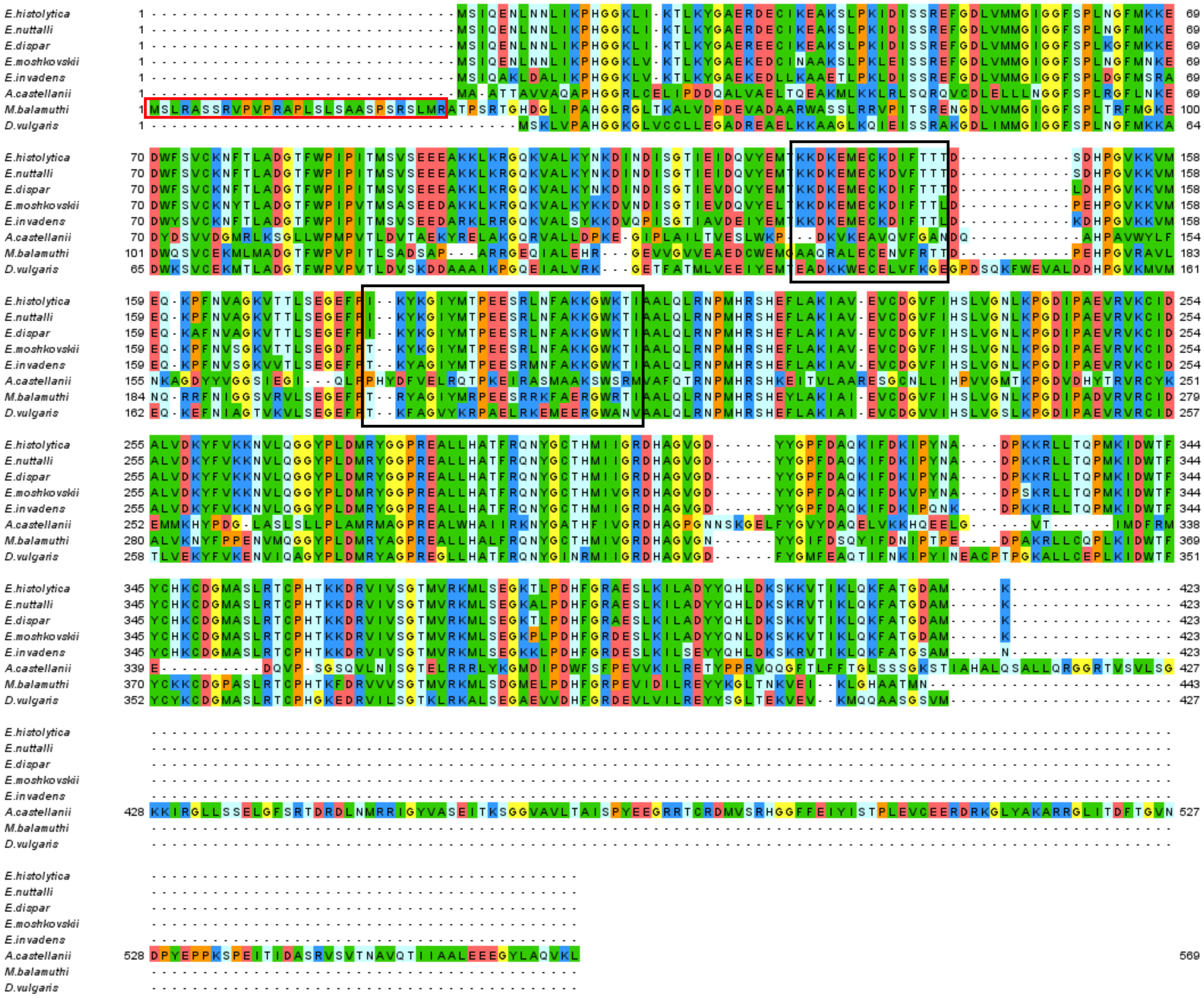

Figure S1

Multiple sequence alignment of ATP sulfurylase of various *Entamoeba* species namely, *Entamoeba histolytica, E. nuttalli, E. dispar, E. moshkovskii*, and *E. invadens*, as well as that of *Acanthamoeba castellanii, Mastigamoeba balamuthi*, and *Desulfovibrio vulgaris*. The figure was created using Clustal Omega with the default parameters [1]. The alignment was displayed using Jalview [2]. The hydrophobic, positively charged, negatively charged, hydrophilic, glycine and proline residues are colored green, blue, red, light blue, yellow and orange respectively. Red box indicate the N-terminal targeting sequence of *Mb*AS predicted by MitoFates [3]. Black boxes indicate the position of the two internal targeting sequence in *Eh*AS.

microorganisms

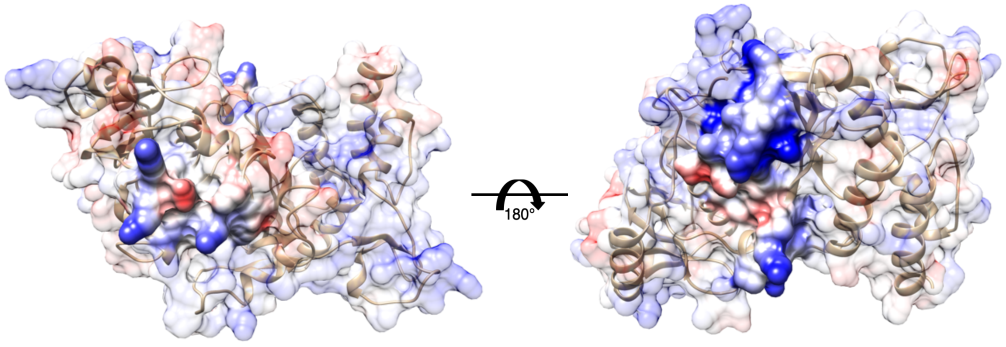

(a)

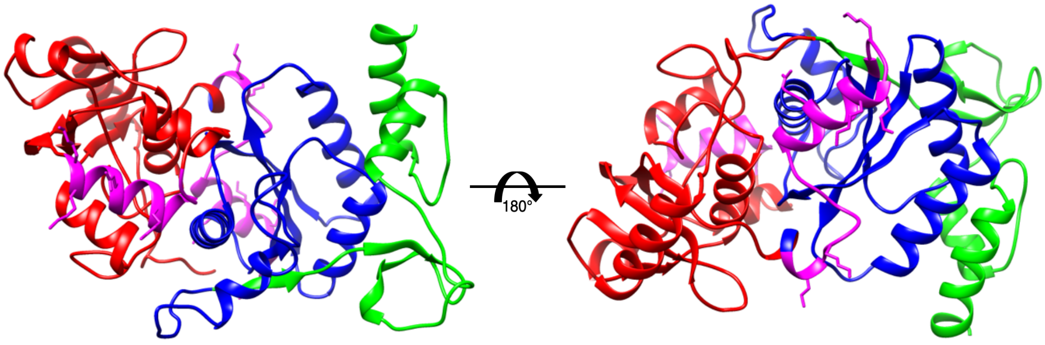

(b)

Figure S2

Structural features of *Eh*AS internal targeting sequences. (a) Surface charge of *Eh*AS. Positively (blue) and negatively (red) charged surfaces of both sides, including two ITS segments: 133-147:KKDKEMECKDIFTTT (left panel) and 179-203:IKYKGIYMTPEESRLNFAKKGWKTI (right panel), of *Eh*AS are depicted. The surface, other than two identified regions, of AS is transparent. (b) The locations and structures of two ITS segments in *Eh*AS. Two ITS segments: 133-147:KKDKEMECKDIFTTT (left panel) and 179-203:IKYKGIYMTPEESRLNFAKKGWKTI (right panel) and their surface lysine and arginine residues are shown in magenta. Three blocks are indicated by red, blue, and green, respectively.

Reference:

1. Sievers, F.; Wilm, A.; Dineen, D.; Gibson, T.J.; Karplus, K.; Li, W.; Lopez, R.; McWilliam, H.; Remmert, M.; Söding, J.; et al. Fast, scalable generation of high-quality protein multiple sequence alignments using Clustal Omega. *Mol. Syst. Biol.* **2011**, *7*, doi:10.1038/msb.2011.75.
2. Waterhouse, A.M.; Procter, J.B.; Martin, D.M.A.; Clamp, M.; Barton, G.J. Jalview Version 2-a multiple sequence alignment editor and analysis workbench. *Bioinformatics* **2009**, *25*, 1189–1191, doi:10.1093/bioinformatics/btp033.
3. Fukasawa, Y.; Tsuji, J.; Fu, S.-C.; Tomii, K.; Horton, P.; Imai, K. MitoFates: Improved Prediction of Mitochondrial Targeting Sequences and Their Cleavage Sites. *Mol. Cell. Proteomics* **2015**, *14*, 1113–1126, doi:10.1074/mcp.M114.043083.
